# A flowchart for adequate controls in virus-based monosynaptic tracing experiments identified Cre-independent leakage of the TVA receptor in RΦGT mice

**DOI:** 10.1186/s12868-024-00848-1

**Published:** 2024-02-21

**Authors:** Anna Velica, Klas Kullander

**Affiliations:** Department of Immunology, Genetics and Pathology, Uppsala University, 815, Husargatan 3, Uppsala, 751 08 Sweden

**Keywords:** TVA leakage, Retrograde monosynaptic viral tracing, RΦGT mice, Flowchart control experiment, G-protein, Retrograde, Rabies virus, Helper virus

## Abstract

**Background:**

A pseudotyped modified rabies virus lacking the rabies glycoprotein (G-protein), which is crucial for transsynaptic spread, can be used for monosynaptic retrograde tracing. By coupling the pseudotyped virus with transgene expression of the G-protein and the *avian leukosis and sarcoma virus subgroup A receptor* (TVA), which is necessary for cell entry of the virus, researchers can investigate specific neuronal populations. Responder mouse lines, like the RΦGT mouse line, carry the genes encoding the G-protein and TVA under Cre-dependent expression. These mouse lines are valuable tools because they reduce the number of viral injections needed compared to when using helper viruses. Since RΦGT mice do not express Cre themselves, introducing the pseudotyped rabies virus into their brain should not result in viral cell entry or spread.

**Results:**

We present a straightforward flowchart for adequate controls in tracing experiments, which we employed to demonstrate Cre-independent expression of TVA in RΦGT mice.

**Conclusions:**

Our observations revealed TVA leakage, indicating that RΦGT mice should be used with caution for transgene expression of TVA. Inaccurate tracing outcomes may occur if TVA is expressed in the absence of Cre since background leakage leads to nonspecific cell entry. Moreover, conducting appropriate control experiments can identify the source of potential caveats in virus-based neuronal tracing experiments.

## Introduction

Identifying the roles of different brain regions and cell types, as well as the connectivity between them, is an important step toward understanding how information is processed in the brain. Historically, monosynaptic connections have been studied using elaborate and time-consuming techniques such as electrophysiological recordings and electron microscopy. However, a decade ago, a method for viral-based monosynaptic retrograde tracing was established [1]. It is based on a pseudotyped genetically engineered rabies virus vector that lacks the rabies virus glycoprotein (G-protein), which is essential for viral spread and infection. The G-protein is found in the viral membrane [2] and acts as a ligand for several receptors, including nicotinic acetylcholine receptors, neuronal cell adhesion molecules [3], metobotropic glutamate receptor subtype 2 [4] and the mammalian p75 neurotrophin receptor, which is also involved in programmed cell death [3, 5]. The G-protein is important for attachment of the virus to the target cell [3] and essential for retrograde transsynaptic transport of the virus [6]. In the modified rabies viral vector, the membrane surface is pseudotyped with the *avian leukosis and sarcoma virus envelope glycoprotein of subgroup A (EnvA)* instead of the wild-type G-protein. This allows it to infect cells that express the transgenic receptor *Avian leukosis and sarcoma virus subgroup A receptor (TVA)* [7]. The TVA receptor is related to the ligand binding domain of the low-density lipoprotein receptor, however mammalian cells do not endogenously express TVA [8, 9]. Thus, in combination with promoter-specific transgene expression of the G-protein and TVA, injection of the EnvA-coated G-protein deleted rabies virus (RVdG-EnvA), into the region of interest allows for the investigation of input connections to a predefined neuronal population [1].

A transgenic responder mouse line that expresses the G-protein and TVA in a Cre-dependent manner was used in this study. The two transgenes are placed downstream of a ubiquitous promoter followed by a stop cassette flanked with LoxP sites [10]. If the bacterial enzyme Cre is present, the DNA will be recombined at the LoxP sites, and the stop fragment will be removed, leading to G-protein and TVA expression [11]. This mouse tool simplifies virus-based tracing experiments since the transgenic mouse line delivers the needed helper tools, circumventing the need for injection of additional viral vectors carrying the G-protein and TVA [12–15]. Theoretically and ideally, the transgenes should not be expressed in the absence of Cre.

In this study, we developed a workflow to identify potential flaws and necessary controls in neuronal virus-based tracing experiments. Our findings revealed concerns regarding the reliance of the RΦGT mouse line since we found Cre-independent TVA leakage. This highlights the importance of conducting proper control experiments prior to the application of this technique. With our proposed workflow, researchers can ensure the accuracy and reliability of their results when using viral tracing methods in neuroscience research.

## Materials and methods

### Mice

All the experiments were approved by the local Swedish ethical committee (Uppsala djurförsöksetiska nämnd) and followed the Swedish Animal Welfare Act (Svensk författningssamling (SFS) 2018:1192), the Swedish Animal Welfare Ordinance (SFS 2019:66), the Regulations and General Advice for Laboratory Animals (SJVFS 2019:9, Saknr L 150), and the ethical permit number C63/16. Mice were bred and housed according to the regulations and guidelines of Jordbruksverket (ethical permit number C135/14). Female and male adult mice between eight and twenty-five weeks of age were used. All the mice were kept on a C57BL/6 background. In total, two wild-type C57BL/6 (Taconic), seven RΦGT (Jackson Laboratory Stock No: 024708) [10], one Chrna2Cre mouse, produced and bred in our own facility [16, 17], and two Chrna2Cre-RΦGT mice were used (bred from RΦGT and Chrna2Cre mice in our own facility). The Chrna2Cre mice were heterozygous for the transgene, while the RΦGT mice were either heterozygous or homozygous. The animals were housed with littermates (up to five animals/cage), kept on a 12-h light/light off cycle (7 a.m.–7 p.m.), and maintained at 21 ± 2 °C with free access to food and water.

### Polymerase chain reaction

Polymerase chain reaction (PCR) was used to genotype the transgenic mice. Mouse ear biopsies were heated to 96°C in 50 µl of 25 mM NaOH for 25 minutes shaking at 300 rpm on a heating block (Mixing Block MB-102, Sweden). After five minutes of cooling, 50 µl of 40 mM Tris HCl with 2 mM EDTA (Sigma Aldrich, Sweden) was added. One microliter of extracted DNA (approximately 20–100 ng/µl) was added to 19 µl of master mix (15.4 µl of dH2O, 2 µl of KAPA Taq buffer (10×) (Kapa Biosystems, Germany), 0.5 µl of 10 mM dNTPs (Thermo Scientific, Sweden), 0.01 µl of KAPA Taq (Kapa Biosystems, Germany), 0.5 µl of forward primer (Chrna2Cre; 5’-AAGCAAATTTTGGTGTACGG-3’ or RΦGT; 5’-CAATTTGGTAGTGGAGGACG-3’) and 0.5 µl of reverse primer (Chrna2Cre; 5’-AGAACTGG AAAGCAGGATGG-3’ or RΦGT; 5’-CTGGTGTTGGGCGGAAATG-3’) and run in a thermal cycler (Bio-Rad T100 Thermal Cycler, Sweden) according to either program 1 or 2. For program 1, the temperature was first set at 94 °C for two minutes, thereafter at 94 °C for 30 s, 65 °C for 30 s and 68 °C for 30 s. The cycle was repeated ten times, but for each cycle, the temperature was set at 0.5 °C lower for each step of the cycle. After ten cycles, the temperature was set at 94 °C for 30 s, 60 °C for 30 s and 72 °C for 30 s, and the new cycle was repeated another 28 times. Finally, the temperature was set at 72 °C for five minutes and then at 10 °C until the product was loaded on the gel. Program 2 started at 95 °C, and after three minutes, the temperature was set at 95 °C for 18 s, 55 °C for 18 s and 72 °C for 24 s. The cycle was then repeated 30 times before the temperature was set at 72 °C for six minutes, 20 °C for 12 s and lastly at 10 °C until the product was loaded on the gel.

PCR products were loaded in 1% agarose (VWR, Sweden) gel with 0.5 µg/ml ethidium bromide (Sigma Aldrich, Sweden) and run for 30 min at 145 W in TAE buffer (VWR, Sweden) using a Consort EV243 and Bio-Rad Sub-Cell GT apparatus. The bands were visualized with a Benchtop 2UV Transilluminator (UVP Sweden). For both genes, a band of 200 base pairs was considered positive, while the absence of a band was considered negative.

### Surgery

Mice were anesthetized with 1.5-4% isoflurane (Baxter) and placed in a stereotaxic device (Stoelting, USA). An anal thermometer and a heating pad (CMA, Sweden) were used to maintain the body temperature at 37 °C. The eyes were kept moist with carbomer (Oftagel 2.5 mg/g). The skin was disinfected with iodine (Jodopax Vet), and analgesic agents were administered subcutaneously: Marcain 2 mg/kg (Bupivacaine, AstraZeneca), Karpofen 5 mg/kg (Norocarp vet, N-vet) and Buprenorphin 0.1 mg/kg (Vertegesic Vet, CEVA). The scalp was incised with scissors, and 3% hydrogen peroxide (Sigma‒Aldrich) was used to remove muscles and connective tissue. Using the Allen Brain Atlas [18] in combination with the stereotaxic device, a craniotomy (1 mm diameter) was performed at the desired coordinates (AP 0.8, ML 1.15, DV 1.4). A total of 400 nl of SAD-ΔG-RV-GFP (EnvA) (viral titer: 3.5 × 10^8^ virus particles/ml; a gift from Prof. Dr. Conzelmann, LMU Munich, Germany) or SAD-ΔG-RV-mCherry (EnvA) (viral titer: 3.0 × 10^8^ virus particles/ml; a gift from Prof. Dr. Conzelmann, LMU Munich, Germany) was injected at a speed of 100 nl/minute with a 10 µl Nanofil Hamilton syringe (WPI, USA). Immediately after the first injection, another injection of 400 nl of the same virus was performed at the same speed and at the same coordinates. In some cases, 200 nl of AAV2/Ef1a-DIO-EYFP (viral titer: 4.6 × 10^12^ virus particles/ml; Lot# AV4842D; UNC Vector Core) was also injected. Five minutes after the last injection, the needle was withdrawn, and the skin was sutured (Vicryl Rapide 6 − 0, Ethicon, REF W9913). Post-surgery, the mice were monitored with attention given to the humane endpoint scoring system for discomfort and pain.

The modified rabies virus was handled in a designated biosafety 2 injection room, in a class 2 microbiological safety cabinet (Safemate EZ 1.2, Bioair). Access to the biosafety 2 injection room was only granted after completion of a mandatory biosafety 2 course at Uppsala University. Viral aliquots were stored at -80 °C and kept at 4 °C during the day of surgery until injected. All instruments used were cleaned with 10% chlorine followed by 70% Ethanol at the end of the injection day. The experimenter wore a single-use protective coat, a cap, shoe covers and double nitrile gloves (VWR, REF 112–2372) when inside the injection room. Mice were kept in ventilated cages in the biosafety 2 injection room until perfusion.

### Tissue processing and imaging

Seven days post injection, the mice were anesthetized with Medetomidine (1 mg/kg; Dormitor Vet, Orion Pharma Animal Health) and Ketamin (75 mg/kg; Ketalar, Pfizer) and sacrificed by transcardial perfusion, initially with 10 ml of phosphate-buffered saline (PBS; Sigma Aldrich, Sweden), followed by 15 ml of 4% paraformaldehyde (PFA; Histolab, Sweden) diluted in PBS. The brain was dissected and postfixed in 4% PFA overnight (12–24 h).

The entire brain was sectioned on a vibratome (Leica VT 1000s, Sweden) into 100 μm thick sections. The sections were washed in PBS for five minutes, stained with DAPI (Sigma Aldrich, 200 ng/ml) in PBS for ten minutes, washed with PBS for fifteen minutes and then mounted with Prolong Diamond Antifade Mountant (refractive index of cured mountant 1.47; REF P36962, LOT 1,880,704; Invitrogen by Thermo Fisher Scientific, Eugene OR USA) on glass slides. Every fourth section of the brain was analyzed under a wide-field fluorescence microscope (Olympus BX61W1, Sweden) with a 10x (NA 0.40) objective. The images were acquired with a 4x (NA 0.16) objective and Volocity 4.1.0 software, and the brightness was adjusted using Fiji software [19].

### Cell counts

The coronal section with the highest number of traced cells at the injection site was chosen for each animal. The cells were counted manually using Fiji software [19]. Statistical analyses were performed using R Statistical Software version 4.0.0 (R Foundation for Statistical Computing, Vienna, Austria). A Kruskal‒Wallis rank sum test with a significance level of 0.05 was used to investigate the effects of mouse genotype (Chrna2Cre-RΦGT vs. RΦGT) on outcome (number of traced cells).

## Results

**Fig. 1:**
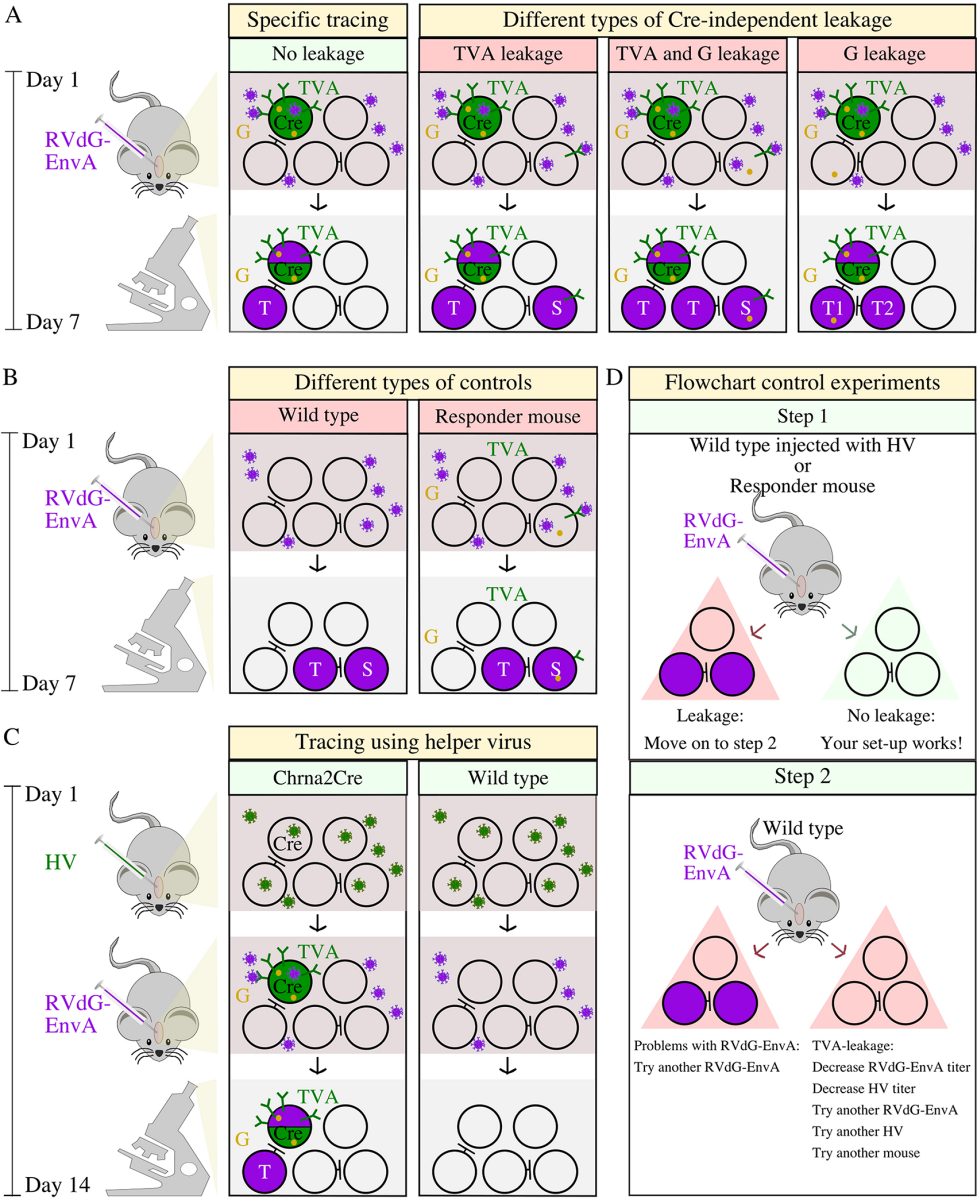
Problems that can occur in monosynaptic retrograde tracing and a flowchart to control for them. (**A**) The aim of monosynaptic retrograde viral tracing experiments is to identify presynaptic connections (purple) to a pre-determined population of neurons (green). If there is no leakage of the Cre-dependent transgenes TVA (green receptor) and G-protein (yellow dot), the RVdG-EnvA will exclusively enter starter cells expressing Cre (Cre) and subsequently spread retrogradely one synapse. If there is TVA-leakage, the RVdG-EnvA can also infect cells lacking Cre. In cases of both TVA and G-protein leakage, there will be an increase in both starter cells (S) and traced cells (T). If there is only G-protein leakage, the possibility of additional traced cells (T2) arises if the G-protein is expressed in presynaptic cells (T1) that target the starter cells (Cre). (**B**) Injecting the RVdG-EnvA into wild-type mice serves as a control for the specificity of the RVdG-EnvA, while injecting it into responder mice carrying the G-protein and TVA under Cre-dependent expression serves as a control for evaluating potential leakage of TVA and G-protein. If there are labelled starter cells (S) after injection of the RVdG-EnvA into wild type mice, the RVdG-EnvA can enter cells independent of EnvA-TVA binding. If there are labelled starter cells (S) after injection of the RVdG-EnvA into responder mice, there might be TVA-leakage. The presence of traced cells (T) suggests G-protein leakage. (**C**) Illustration of tracing experiments using helper virus (HV). The HV enters most cells, but TVA and G-protein are expressed in a Cre-dependent manner. One-week post injection of HV the RVdG-EnvA is injected infecting cells expressing TVA and spreading from cells expressing G-protein. (**D**) Flowchart with proposed control experiments for monosynaptic retrograde tracing. Red colour in title boxes indicates a problem, whereas green colour indicates desired result

To validate the effectiveness of our tracing method, we first developed a comprehensive flowchart for conducting appropriate control experiments. The RVdG-EnvA is coated with the EnvA ligand, ideally allowing it to enter only cells expressing TVA. Additionally, the virus lacks the G-protein necessary for viral spread, meaning that it should only spread from cells expressing the G-protein. In the offspring of responder mice (which carry the G-protein and TVA under Cre-dependent expression) bred with Cre-driver mice, the G-protein and TVA should only be expressed in Cre-positive cells. However, if the virus is found in Cre-negative cells, it may be due to EnvA-TVA-independent cell entry or due to Cre-independent TVA expression allowing cell entry (Fig. 1A). If injection of RVdG-EnvA into the region of interest of wild-type mice results in traced cells, the RVdG-EnvA itself is nonspecifically infecting cells that lack TVA. However, if there are traced cells after injection of the RVdG-EnvA in responder mice, this could be due to nonspecific RVdG-EnvA infection, as in the prior example, or due to Cre-independent leaky expression of TVA (Fig. 1B). Conclusively, control injections of RVdG-EnvA into the region of interest in wild-type mice do not account for TVA leakage.

To account for G-protein and TVA leakage and ensure the accuracy of the results, the RVdG-EnvA should be injected into the region of interest in responder mice, which carry the G-protein and TVA under Cre-dependent expression, to conduct a blank control injection. Likewise, if helper virus (HV) transgene delivery of the G-protein and TVA is used (Fig. 1C), RVdG-EnvA should be injected into wildtype mice previously injected with the helper virus. If the control injection produces no traced cells, no further control experiments are necessary. However, if traced cells are present, additional control experiments must be conducted to determine the cause and guide appropriate adjustments or protocol optimizations (Fig. 1D).

**Fig. 2:**
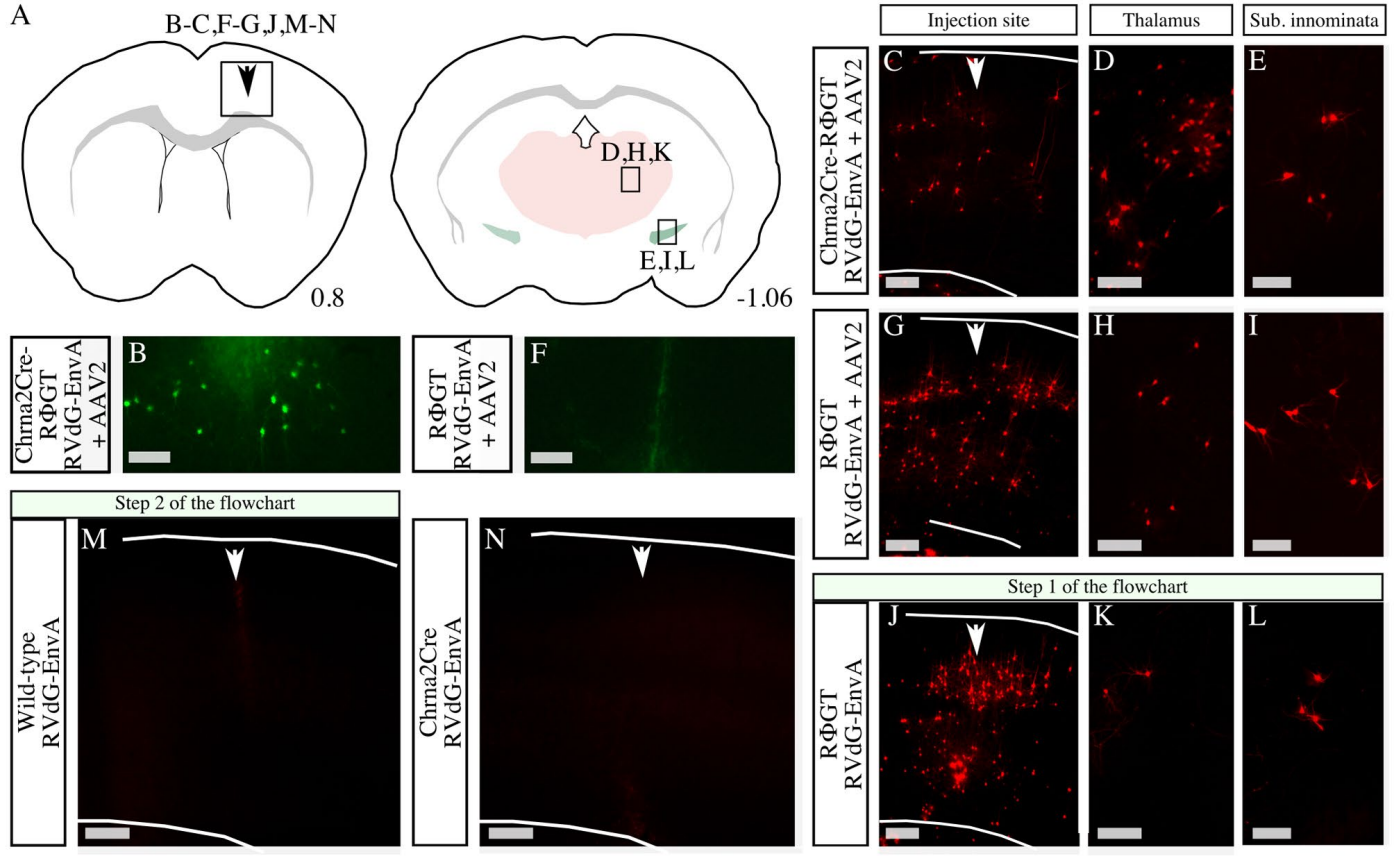
RΦGT mice express TVA independent of Cre protein presence. (**A**) Schematic illustration of the brain regions with observed traced cells. Traced cells were found in the cortex by the injection site (arrow) at bregma 0.8, as well as in the thalamus (red) and substantia innominata (green) at bregma − 1.06. Letters refer to the location from which the micrographs in the figure are imaged. (**B-E**) Micrographs from Chrna2Cre-RΦGT mice injected with a Cre-dependent EYFP expressing adeno-associated viral vector serotype 2 (AAV2) and a mCherry expressing modified G-protein deleted EnvA coated rabies virus (RVdG-EnvA). Arrow points at the injection site, lines mark the upper and lower borders of the cortex. There were EYFP positive cells (**B**) and mCherry positive wells by the injection site (**C**), as well as mCherry positive cells in thalamus (**D**) and substantia innominata (**E**). (**F-I**) Micrographs from RΦGT mice injected with AAV2 and RVdG-EnvA. Arrow points at the injection site, lines mark the upper and lower borders of the cortex. There were no EYFP positive cells (**F**) at the injection site. However, mCherry positive cells were found by the injection site (**G**) as well as in the thalamus (**H**) and substantia innominata (**I**). (**J-L**) Micrographs from RΦGT mice injected with RVdG-EnvA. Arrow points at the injection site, lines mark the upper and lower borders of the cortex. There were mCherry positive cells by the injection site (**J**) as well as in the thalamus (**K**) and substantia innominata (**L**). (**M-N**) Micrographs of the cortex of wild type (**M**) and Chrna2Cre (**N**) mice injected with RVdG-EnvA. Arrow points at the injection site, lines mark the upper and lower borders of the cortex. No traced cells were observed. Scale bars: 100 μm in D and H, 200 μm in B-C and E-N

In this study, we used the RΦGT mouse line for transgene expression of the G-protein and TVA. To assess the ability of the viral tracer to infect and spread throughout the brain, we injected an adeno-associated virus serotype 2 (AAV2) carrying an *enhanced yellow fluorescent protein (EYFP)* and a mCherry-expressing RVdG-EnvA into the cortex of Chrna2Cre-RΦGT mice (*n* = 2). AAV2 can enter any cell near the injection site, however EYFP is expressed in a Cre-dependent manner. In contrast, RVdG-EnvA only infects TVA-expressing cells, and mCherry is expressed in all RVdG-EnvA-infected cells. Several cells near the injection site were indeed positive for EYFP (Fig. 2A-B), and there were also mCherry-positive traced cells at the injection site (Fig. 2C). Furthermore, traced cells were found in remote brain regions such as the thalamus and substantia innominata (Fig. 2D-E). Hence, the RVdG-EnvA can effectively infect TVA-expressing cells and trace their connected presynaptic cells in distant brain regions.

To ensure the reliability of the tracing method, we conducted control experiments. We injected the EYFP-expressing AAV2 and the mCherry-expressing RVdG-EnvA into the cortex of RΦGT mice (*n* = 3). As expected, no green fluorescent cells were detected, indicating that the AAV2 was exclusively expressed in Cre + cells (Fig. 2F). However, mCherry-positive cells were found near the injection site (Fig. 2G) and in remote brain regions such as the thalamus and substantia innominata (Fig. 2H-I). These results demonstrated that the RVdG-EnvA could infect cells in RΦGT mice in the absence of the Cre. However, AAV2 can initiate receptor-mediated endocytosis through multiple neuronal receptors [20, 21]. Upon endocytosis, the cell membrane absorbs external material located in the proximity of the ligand that initiated the internalization. Thus, the possibility of AAV2-facilitated RVdG-EnvA cell entry cannot be excluded because some RVdG-EnvA particles might be internalized during AAV2-initiated endocytosis.

To determine the cause of the observed mCherry-postive cells in the RΦGT mice, and exclude AAV2-facilitated receptor-mediated endocytosis as a mechanism, we conducted additional control experiments according to the flowchart, beginning with step 1 (Fig. 1D). Specifically, we injected the RVdG-EnvA alone into the cortex of RΦGT mice (*n* = 5). Traced cells were observed both at the injection site (Fig. 2J) and in remote brain regions such as the thalamus and substantia innominata (Fig. 2K-L) in all the injected mice, indicating that the RVdG-EnvA can enter neurons in RΦGT mice independently of AAV2 presence.

To further investigate the significance of TVA leakage in RΦGT mice, we performed cell counts on coronal brain sections from mice injected with RVdG-EnvA (Table 1). A comparison of the number of traced cells after injection of the mCherry-expressing RVdG-EnvA (with or without EYFP-expressing AAV2) revealed no significant difference in the number of traced cells at the injection site in sections from Chrna2Cre-RΦGT mice (158 cells on average, SD = 38, *n* = 2) compared to sections from RΦGT mice (280 cells on average, SD = 247, *n* = 5) (Kruskal‒Wallis rank sum test, data: number of cells by genotype, Kruskal‒Wallis chi squared = 0.6, df = 1, p-value = 0.4386).

**Table 1:** Mice, viruses and cell counts

Animal ID	Sex	Age (weeks)	Strain	Injected virus	Traced cells
56,945	Male	10	Chrna2Cre- RΦGT lx/wt	RVdG-EnvA (mCherry) + AAV2 (EYFP)	185 cells
56,946	Male	10	Chrna2Cre- RΦGT lx/wt	RVdG-EnvA (mCherry) + AAV2 (EYFP)	131 cells
61,096	Female	13	RΦGT lx/lx	RVdG-EnvA (mCherry) + AAV2 (EYFP)	697 cells
61,076	Male	8	RΦGT lx/wt	RVdG-EnvA (mCherry) + AAV2 (EYFP)	223 cells
56,402	Male	23	RΦGT lx/wt	RVdG-EnvA (mCherry) + AAV2 (EYFP)	156 cells
61,097	Female	13	RΦGT lx/lx	RVdG-EnvA (mCherry)	269 cells
56,758	Female	9	RΦGT lx/wt	RVdG-EnvA (mCherry)	55 cells
56,758	Female	9	RΦGT lx/wt	RVdG-EnvA (GFP)	70 cells
56,653	Female	25	RΦGT lx/lx	RVdG-EnvA (GFP)	53 cells
56,650	Female	25	RΦGT lx/lx	RVdG-EnvA (GFP)	67 cells
53,574	Male	15	C57BL/6	RVdG-EnvA (mCherry)	0 cells
53,574	Male	15	C57BL/6	RVdG-EnvA (GFP)	0 cells
57,789	Female	25	C57BL/6	RVdG-EnvA (mCherry)	0 cells
57,789	Female	25	C57BL/6	RVdG-EnvA (GFP)	0 cells
56,642	Female	20	Chrna2Cre	RVdG-EnvA (mCherry)	0 cells
56,642	Female	20	Chrna2Cre	RVdG-EnvA (GFP)	0 cells

The mice and viruses used are presented in this table. The brain section with the highest number of cells at the injection site was chosen for each mouse, and the cell counts are presented in the table

lx/lx = homozygote for TVA

lx/wt = heterozygote for TVA

Following step 2 of the flowchart, we injected the RVdG-EnvA into the cortex of wild-type mice (*n* = 4) and found no traced cells in any of the injected mice (Fig. 2M). This finding suggested that the EnvA-TVA-dependent cell entry of the RVdG-EnvA is highly specific. We then injected the virus into the cortex of Chrna2-Cre mice (*n* = 2) and once again found no traced cells, demonstrating that Cre expression alone does not facilitate viral cell entry (Fig. 2N).

## Discussion

To accurately dissect neuronal circuits, tools with high specificity are essential. Unfortunately, the use of unspecific tools can lead to unreliable tracing results. To address this issue and aid future experimenters, we created a flowchart that outlines common errors in tracing experiments and proposed control experiments to avoid unreliable outcomes (Fig. 1). Following the flowchart, we observed numerous traced cells near the injection site in RΦGT mice that were injected with RVdG-EnvA (Fig. 1D, Step 1). In contrast, no traced cells were found in wild-type mice (Fig. 1D, Step 2). In addition to conducting experiments with a mCherry-expressing RVdG-EnvA, we also performed control experiments with a green fluorescent protein (GFP)-expressing RVdG-EnvA with similar results (Table 1). The absence of traced cells in wild-type mice suggested that the RVdG-EnvA did not enter cells that did not express TVA. Hence, the numerous traced cells found in RΦGT mice revealed Cre-independent leaky expression of TVA (Fig. 1D). Moreover, cell counts from coronal brain sections with traced mCherry-positive cells at the injection site in RΦGT mice indicated that the number of traced cells might be greater in RΦGT mice that are homozygous for TVA than in mice that are heterozygous for TVA (Table 1). However, the small sample size makes it difficult to draw forceful conclusions regarding this matter. The differences in the number of traced cells observed at the injection site in different mice could also be due to technical problems such as clogged injection needles, which can lead to injection of smaller volumes than intended in some cases. Nonetheless, the lack of a significant difference in the number of traced cells at the injection site after RVdG-EnvA injection in Chrna2Cre-RΦGT mice compared to RΦGT mice demonstrates the potential problem of using the RΦGT mouse line for EnvA-TVA-dependent tracing experiments and emphasizes the need for adequate control experiments in tracing experiments to obtain reliable results.

The observed traced cells in distant brain regions of RΦGT mice injected with RVdG-EnvA indicated either axonal “leaky” expression of TVA in presynaptic cells followed by retrograde intracellular transport of the virus or transsynaptic spread of the virus from initially infected starter cells to presynaptic cells due to G-protein leakage [12, 22, 23]. In the original publication, injection of G-protein-coated G-protein-deleted rabies virus into the mystacial pad musculature of RΦGT mice did not lead to nonspecific long-range projection tracing [10]. This excluded G-protein leakage and nonspecific transsynaptic spread of that particular G-protein-coated rabies virus when it was injected into the mystacial pad musculature. However, potential TVA leakage was not tested [10]. Due to the high affinity of the TVA receptor for EnvA, even low levels of TVA can lead to viral cell entry and transgene expression [24]. For some TVA variants, the binding of a single virion to a single TVA receptor is sufficient for cell entry initiation [25]. In contrast, higher levels of G-protein are needed for viral transsynaptic spread [24]. Our results show TVA, and possibly G-protein, leakage in RΦGT mice. Even if TVA leakage is sufficient to affect the results, possible G-protein leakage might not be strong enough to lead to retrograde transsynaptic spread of the viral tracer.

The information provided by Jackson laboratories regarding the RΦGT mouse line is misleading since it states [26]: *“Expression of RABVgp4 and TVA is blocked by a loxP-flanked STOP fragment placed between the construct sequence and the Gt(ROSA)26Sor promoter.”* However, our findings indicate that TVA leakage can still occur despite this claim. Furthermore, Jackson laboratories state: *“In 2023, the RΦGT allele in Stock No. 024708 was discovered to have always had a 1 bp insertion immediately following the TVA start codon that causes a frameshift and the resulting protein is predicted to be non-functional”.* However, our findings indicate that the TVA protein is functional in the absence of Cre in the motor cortex of RΦGT mice. It is important to note that several studies have successfully used the RΦGT mouse line for viral monosynaptic retrograde neuronal tracing, despite this issue [27]. There are several possible explanations for this. First, RΦGT mice might have a functional TVA in some genome regions but not in others. Second, viral vectors originating from different rabies virus strains, such as the SAD-B19 vaccine strain [1] and the CVS-N2c(ΔG) strain [28], might have different properties affecting the results. The CVS-N2c(ΔG) strain has a lower neuronal toxicity and enhanced transsynaptic transfer, compared to the SAD-B19 vaccine strain, but it is more complicated to produce, as it requires longer preparation cycles and has a lower yield [28]. However, recently improved production systems, yielding higher titers at lower production times, have been developed, making the CVS-N2c(ΔG) virus more appealing in tracing studies [29, 30]. There are also rabies viral tracers with additional deleted viral genes with potentially lower risk of nonspecific transsynaptic spread [31]. Third, the viral titer, injection volume and incubation time might impact the results [32]. Therefore, we believe that it is crucial to perform control experiments before using a rabies viral tracer in a new mouse line or a new target area. In any case, many studies that have successfully used the RΦGT mouse line have not used the EnvA-TVA system but instead focused on injecting G-protein-coated rabies viral vectors [33–37]. Notably, in some of the previous studies, including several landmark papers, EnvA-coated rabies viral vectors were used with no control for Cre-independent TVA leakage. The authors relied on the EnvA-TVA system for the experiments but did not inject the RVdG-EnvA into RΦGT mice to control for TVA leakage. Some studies did not perform any control experiments for retrograde tracing [38–41], while others controlled only for EnvA-TVA-independent cell entry by injection in wild-type mice [42]. Others have only controlled for G-protein leakage by injecting G-protein-coated rabies viral tracers into RΦGT mice [10, 43]. Alternatively, some studies compared tracing results between different RΦGT;Cre-lines with no control injection in RΦGT mice [44]. Despite the limited sample size in our study, we believe that the results showing leaky expression of TVA in the RΦGT mouse line can benefit the neuroscience community, and that the flowchart for adequate control experiments can guide future researchers in their work with viral tracers.

To achieve transgene expression of the G-protein and TVA, viral delivery through helper viruses is also commonly used [12–15]. Using a viral vector for transgene introduction of the G-protein and TVA allows for titration of the viral titer and transgene expression to minimize transgene leakage [22]. Additionally, the use of mutated TVA variants minimizes the consequences of possible TVA leakage [45]. However, when using a helper virus, it is necessary to perform controls for Cre-independent leakage of the helper virus itself, in addition to performing control injections of the modified rabies virus alone (Fig. 1D).

In summary, our study revealed TVA leakage in the RΦGT mouse line, which can lead to unspecific results when using rabies viral tracers. Our aim was not to provide a full characterization of the TVA leakage in the RΦGT mouse line. Rather, we wish to highlight a potential problem with the RΦGT mouse line and emphasize the need to conduct rigorous control experiments when using viral tracers. We hope that our findings will encourage future scientists to control for G-protein and TVA leakage, for example by the use of the supplied flowchart, before using viral tracers in a new mouse line or a new target region, to reach robust and reliable conclusions from their tracing analyses.

## Conclusion

Following a simple flowchart for adequate control experiments in virus-based tracing experiments minimizes the risk of inaccurate tracing results. By following the proposed flowchart, we revealed Cre-independent TVA leakage in the RΦGT mouse line.

## Data Availability

The datasets used and/or analysed during the current study are available from the corresponding author on reasonable request.

## Abbreviations

AAV2: adeno-associated virus, serotype 2
EnvA: avian leukosis and sarcoma virus envelope glycoprotein of subgroup A
EYFP: enhanced yellow fluorescent protein
G-protein: rabies virus glycoprotein
GFP: green fluorescent protein
RVdG-EnvA: EnvA-coated G-protein deleted rabies virus
TVA: avian leukosis and sarcoma virus subgroup A receptor
